# Synergistic Charge Scavenging in Pt/C‐Polyoxometalate Systems for Scalable Biomass Power Generation via Super‐Reduced Electrolytes

**DOI:** 10.1002/advs.78361

**Published:** 2026-10-25

**Authors:** Hojin Lee, Hyeong‐Jin Lee, Jongseo Lee, Sang‐Soo Han, Sangjin Park, Eun Seon Cho, Hee‐Tak Kim

**Affiliations:** ^1^ Department of Chemical and Biomolecular Engineering Korea Advanced Institute of Science and Technology (KAIST) Daejeon Republic of Korea; ^2^ Clean Energy Research Center Korea Institute of Science and Technology (KIST) Seoul Republic of Korea; ^3^ 3rd R&D Institute Agency for Defense Development Daejeon South Korea

**Keywords:** biomass, biomass fuels, electricity generation, electrochemistry, electrolyte, electron transfer, energy harvesting, fuel cells, lignocellulosic biomass, nanotechnology, polyoxometalate, power density

## Abstract

Direct electrochemical conversion of unrefined biomass waste into electricity offers a sustainable pathway for decentralized energy generation, yet its practical implementation has been fundamentally hindered by low power densities and limited scalability. A fundamental bottleneck lies in the intrinsic recalcitrance of biomass, which restricts electron harvesting to shallow oxidation pathways and confines redox mediators to low reduction states. Here, we demonstrate a scalable biomass flow fuel cell (BFFC) enabled by a super‐reduced polyoxometalate (POM) electrolyte that overcomes the long‐standing electron‐harvesting ceiling. By employing a catalytic oxidative degradation strategy that promotes extensive cleavage of recalcitrant C─C linkages in the lignocellulosic matrix, we unlock deep multi‐electron reduction of POM, achieving an electron transfer capacity exceeding four electrons per molecule. As a result, the single‐cell device delivers a record‐breaking power density of 218.6 mW cm^−^^2^. Beyond laboratory‐scale demonstrations, we constructed a 10‐cell stack prototype, producing 59.2 Wh over 15.6 h of stable operation directly from raw biomass.

## Graphical abstract

Synergistic charge scavenging between Pt/C and liquid polyoxometalate mediators effectively dismantles the inherent biological recalcitrance of raw biowaste. This dynamic interfacial electron transfer unlocks a multi‐electron super‐reduced state (>4 electrons). By overcoming traditional electron‐harvesting ceilings, this approach enables scalable, high‐power electricity generation directly from unpurified biomass within a flow fuel cell.

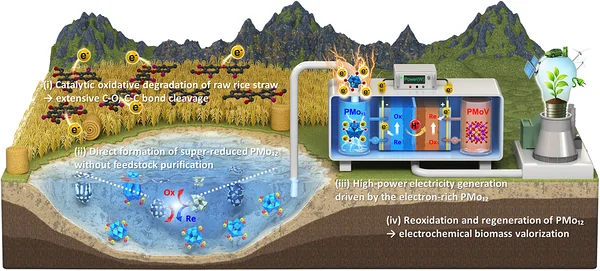

## 1 Introduction

The global transition toward a carbon‐neutral economy necessitates the efficient valorization of abundant but underutilized fuels such as biomass waste as a sustainable and clean energy source [1, 2]. Currently, biomass utilization, dominated by traditional combustion, is fundamentally restricted by Carnot cycle limits and energy‐intensive drying pretreatments that cause massive exergy losses and severe CO_2_ emissions. Alternatively, catalytic or electrochemical biomass valorization and energy extraction offer a highly sustainable and decentralized paradigm [3, 4, 5]. Specifically, biomass flow fuel cells (BFFCs), which directly convert raw biomass into electricity, offer an environmentally benign and highly efficient pathway for biomass utilization and power production [6]. By operating at low temperatures (< 100°C) in aqueous media, BFFCs entirely bypass costly pretreatments while providing superior conversion efficiencies free from thermal thermodynamic limits. Despite these compelling macroscopic advantages, the practical implementation of BFFCs remains severely hindered by low power densities and limited scalability, primarily due to the slow oxidative degradation and incomplete decomposition of biomass waste components [7]. A fundamental bottleneck lies in the intrinsic recalcitrance of carbon‐based bonds in raw biomass.

As a representative type of BFFC device, polyoxometalate (POM)‐based BFFCs, which use POMs as both organic or biomass degradation mediators and electron carriers, are the most widely employed [8, 9, 10, 11, 12, 13, 14, 15, 16, 17, 18]. Since POM‐based electrolytes are used at both the anode and the cathode, the system offers advantages in mitigating ion crossover, but a fundamental limitation persists: the recalcitrant lignocellulosic matrix strongly restricts the extent of oxidative bond cleavage, confining electron extraction to partial oxidation pathways [19, 20]. As a result, the reduction state of POM electrolytes typically remains shallow, and the electron‐storage capacity of the mediator is rarely fully exploited. This intrinsic electron‐harvesting ceiling imposed by biomass recalcitrance has severely capped the attainable voltage output and power density of POM‐driven BFFCs, presenting a key barrier to practical deployment.

Here, we propose that overcoming this bottleneck requires unlocking deep multi‐electron reduction chemistry of POM electrolytes through catalytic restructuring. Specifically, we demonstrate that synergistic catalytic oxidative degradation—combining POM acidity, sulfuric‐acid‐assisted hydrolysis, and Pt/C‐enabled C─C bond scission—can transform raw rice straw into a highly oxidizable fragment pool capable of driving POMs into a super‐reduced redox regime (> 4e^−^ storage per molecule). This catalytic redox‐state engineering strategy enables POM electrolytes to function as practical electron reservoirs rather than conventional mediators, thereby fundamentally shifting the operating regime of BFFCs toward high power and scalable electricity generation.

Using mechanistic analyses based on 2D HSQC NMR and solid‐state ^1^ ^3^C NMR, we clarify the distinct roles of acid and Pt catalysts in selectively cleaving ether/ester and recalcitrant C─C linkages, respectively. Crucially, we reveal that the POM matrix functions as a dynamic electron scavenger, seamlessly capturing accumulated surface electrons from Pt/C. This synergistic charge transfer prevents rapid catalyst poisoning and ensures highly sustainable catalytic activity, directly correlating the resulting bond‐cleavage pathways with the achievable POM reduction states. Leveraging this mechanistic understanding, we realize a record single‐cell power density of 218.6 mW cm^−^^2^. Importantly, we further demonstrate scalability by constructing a 10‐cell stack prototype (total active area 250 cm^2^), which produces 59.2 Wh of electricity over 15.6 h of stable discharge using raw rice straw without purification. These results establish a scalable platform for converting agricultural waste into practical electrical power by breaking the long‐standing trade‐off between biomass recalcitrance and electrochemical energy output.

## 2 Results and Discussion

### 2.1 PMo_12_ Reduction via Catalytic Oxidative Degradation of Raw Rice Straw

We identified that raw rice straw outperforms purified lignin due to its abundant degradable bonds within the lignocellulosic matrix (Figure S1). Yet, utilizing PMo_12_ alone imposes a fundamental limit, confining the electrolyte to a shallow reduction state and leaving its capacity largely unexploited. Since an extensive reduction state is a prerequisite for high power efficiency, breaking this ‘electron‐harvesting ceiling’ required a catalytic breakthrough. Consequently, we implemented a catalytic oxidative degradation strategy utilizing a synergistic system of Pt/C and H_2_SO_4_ to maximize the output.

Several previous studies have reported that platinum or sulfuric acid can enhance the electrocatalytic oxidation of C─O and C─C bonds, which are key structural elements of rice straw [21, 22, 23]. The reduced PMo_12_ solution after 12 h of oxidative degradation of rice straw (Figure S25) in PMo_12_, PMo_12_–Pt/C, PMo_12_–2 m H_2_SO_4_, and PMo_12_–Pt/C–2 m H_2_SO_4_ was investigated using UV–Vis analysis (Figure 1a and Figures S2 and S27). The concentration of sulfuric acid was set to 2 m to prevent precipitation in the PMo_12_ solution (Figure S3). Upon addition of H_2_SO_4_ to the PMo_12_ solution, an increase in absorbance at 650–700 nm—corresponding to the 2‐electron reduced PMo_12_ species—was observed. In contrast, the addition of Pt/C not only intensified this 650–700 nm peak but also introduced a new, pronounced absorption peak near 850 nm, indicative of further reduction. When both Pt/C and H_2_SO_4_ were incorporated, the absorbance at both 650–700 nm and 850 nm significantly increased, indicating a synergistic enhancement in the reduction state of PMo_12_. These trends were further supported by ^3^ ^1^P NMR analysis (Figure 1b), which directly reflects the number of electrons transferred to Mo(VI) [24]. In the case of rice straw decomposition using PMo_12_ alone, only two peaks at −6.07 and −6.73 ppm were observed, corresponding to the 2‐electron reduced state. Upon introducing H_2_SO_4_, a new peak at −13.07 ppm emerged, characteristic of the 4‐electron reduced PMo_12_ species. When Pt/C was used, the intensity of the 4‐electron peak increased markedly alongside the existing 2‐electron peaks. Notably, the combination of Pt/C and H_2_SO_4_ resulted in dominant peaks corresponding to both 2‐ and 4‐electron reduced species, clearly confirming an enhanced reduction state. The presence of the new 850 nm UV–Vis peak in the PMo_12_‐Pt/C system is thus validated as the spectral signature of the 4‐electron reduced PMo_12_ species, consistent with the ^3^ ^1^P NMR data. The calculated reduction degree also showed a consistent trend with the observed electron reduction peaks (Figure 1c). PMo_12_–2 m H_2_SO_4_, which primarily exhibited 1‐ and 2‐electron reductions, showed a reduction degree of 1.88. In PMo_12_–Pt/C, where both 2‐ and 4‐electron reduction states were prevalent, the reduction degree increased to 2.92. Notably, PMo_12_–Pt/C–2 m H_2_SO_4_, which maximized both 2‐ and 4‐electron reductions, achieved a significantly higher reduction degree of 3.58. It should be noted that these reduction degrees were calculated based on a calibration curve established via standard redox titration under baseline conditions (Figure S26). While this linear regression provides reliable quantification within its calibrated range (up to ∼2.5 e^−^), we acknowledge that estimating higher reduction degrees (e.g., >3.0 e^−^) inherently involves mathematical extrapolation. Therefore, while these extrapolated values and spectral signatures indicate the successful formation of a highly reduced POM reservoir (>4 e^−^), the absolute values entail a degree of analytical uncertainty. Precise quantitative evaluation of this extreme super‐reduced state will necessitate the development of specialized redox titration protocols in future studies.

**FIGURE 1:**
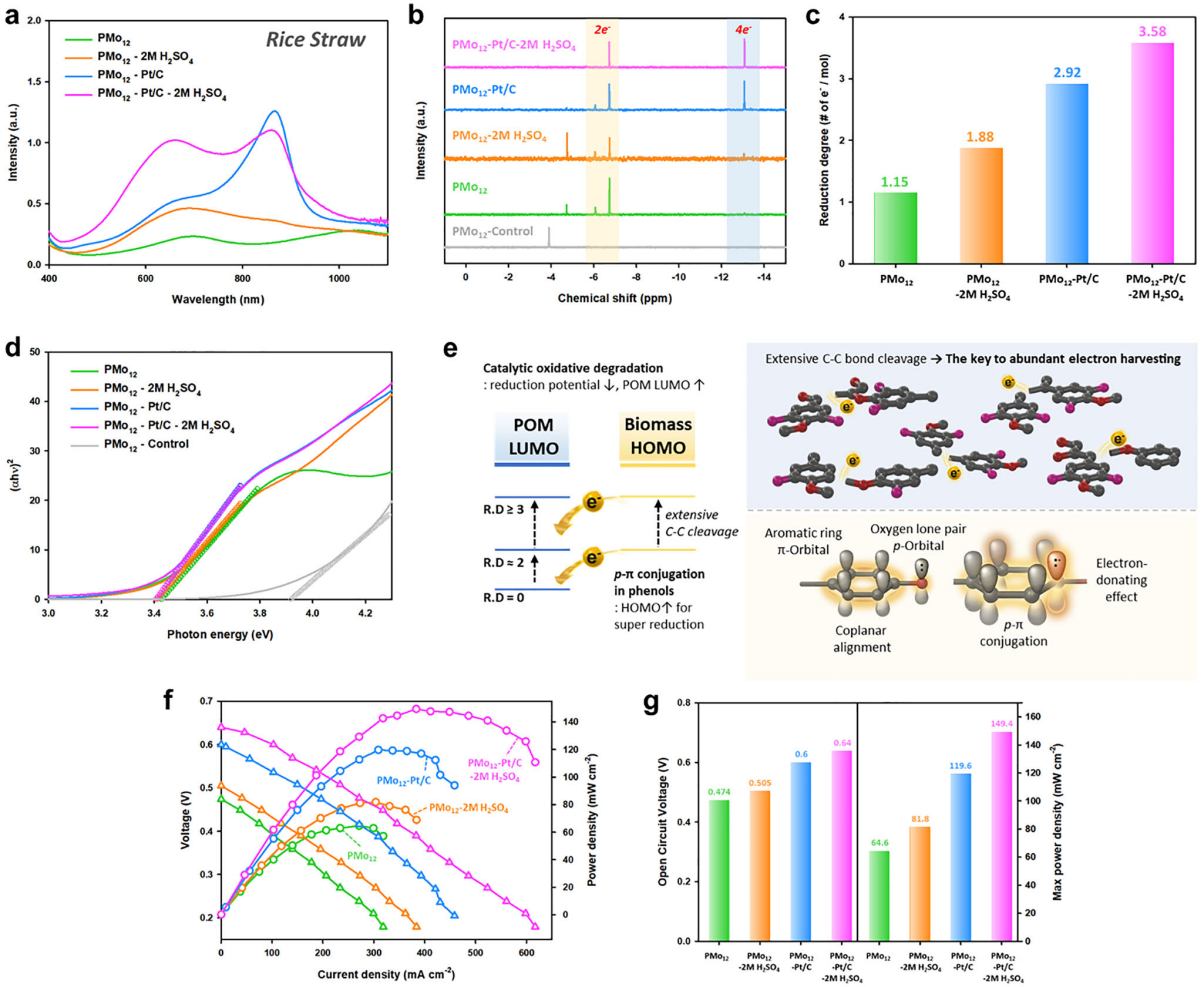
Multi‐electron reduction of PMo_12_ driven by synergistic catalytic oxidative degradation. (a) UV–Vis spectra after catalytic oxidative degradation of raw rice straw in PMo_12_, PMo_12_–2 M H_2_SO_4_, PMo_12_–Pt/C, and PMo_12_–Pt/C–2 m H_2_SO_4_ at 100°C for 12 h. (b) ^3^ ^1^P NMR spectra of the reduced PMo_12_ electrolytes. (c) Reduction degree of PMo_12_ calculated from the UV–Vis spectra after catalytic oxidative degradation. (d) Optical bandgap analyses of the PMo_12_‐control and the reduced samples. (e) Schematic illustration of the thermodynamic mechanism for multi‐electron harvesting. (f) i–V polarization (left *y*‐axis, triangles) and power density (right *y*‐axis, circles) curves of the BFFC using reduced PMo_12_ by catalytic oxidative degradation as anode electrolyte. (g) Open‐circuit voltage (OCV) and maximum power density of the MEAs.

The depolymerization of robust carbon frameworks fundamentally relies on hydrogen atom transfer (HAT) to generate reactive intermediates [25, 26, 27]. Because PMo_12_ alone lacks the capability to initiate this process, our system employs Pt/C to actively drive the HAT process. By efficiently abstracting hydrogen atoms from the biomass, Pt/C substantially lowers the activation energy required for subsequent oxidation steps, fundamentally accelerating the overall macroscopic reaction kinetics [28, 29]. Crucially, the highly concentrated PMo_12_ electrolyte acts as a dynamic electron and proton scavenger, immediately capturing these extracted charge carriers from the Pt/C surface. This seamless synergistic decoupling prevents surface charge accumulation and rapid catalyst poisoning, thereby driving the equilibrium of the HAT and depolymerization reactions strictly in the forward direction [30]. The resulting extensive breakdown liberates abundant electron donors, which in turn drive the multi‐electron reduction of the POM. To elucidate the thermodynamic mechanism underlying the multi‐electron reduction, optical bandgap analyses were conducted (Figure 1d). Compared to the PMo_12_‐control, all reduced samples exhibit a significantly narrowed internal optical bandgap. This narrowing indirectly reflects the progressive filling of the POM's energy states, which inherently elevates the acceptor's effective LUMO level and imposes a severe thermodynamic ceiling against further reduction. To surmount this ascending barrier, the continuous supply of highly potent electron donors with matching high energy levels is strictly required. In our system, this is achieved through the extensive cleavage of robust C─C bonds within the lignocellulosic matrix. This depolymerization generates phenolic moieties characterized by strong p‐π conjugation between oxygen lone‐pair electrons and aromatic rings. Crucially, we hypothesize that this specific orbital conjugation intrinsically yields new electron donors with substantially elevated HOMO levels. Therefore, as the POM's LUMO rises, we propose that the concurrent generation of these high‐HOMO phenolic species effectively compensates for the energy difference [31]. While direct spectroscopic proof of these transient energy levels requires further investigation, this hypothesized synchronized energy shift provides a plausible thermodynamic model that preserves a favorable charge transfer gap, sustaining both the continuous HAT pathway and the thermodynamic driving force required for extreme multi‐electron reduction (Figure 1e). To experimentally support this hypothesized synchronized energy shift, we systematically evaluated the intrinsic electron‐donating contributions of representative biopolymers and their model monomers (as detailed in Figure S4). The control experiments revealed that the primary monolignol exhibits an exceptional capacity to reduce the POM up to 4.2 e^−^, while sugar‐derived species exhibit moderate electron‐donating capacities (1.4–2.5 e^−^). These results suggest that highly conjugated phenolic moieties serve as the primary high‐HOMO electron donors capable of surmounting the ascending thermodynamic barrier. Furthermore, the highly restricted reduction degree of un‐catalyzed polymeric lignin (0.4 e^−^) highlights the critical role of the Pt/C catalyst. The extensive HAT mechanism driven by Pt/C is essential to effectively deconstruct the recalcitrant matrix, continuously liberating the locked high‐HOMO phenolic species (the 4.2 e^−^ donors) from the bulky polymeric network, thereby enabling the super‐reduction.

The electrochemical performance of the BFFC single cell was evaluated using each of the reduced anode electrolytes (Figure 1f,g). To complete the full‐cell configuration (Figure S28), phosphomolybdovanadate (PMoV)—a well‐established O_2_‐regenerable liquid mediator—was employed as the catholyte. As demonstrated in pioneering flow fuel cell studies [16], utilizing a homologous POM‐based system at both electrodes inherently mitigates irreversible cross‐contamination caused by active material crossover, ensuring long‐term operational stability. The voltage measured at OCV increased in the following order: PMo_12_< PMo_12_–2 m H_2_SO_4_< PMo_12_–Pt/C < PMo_12_–Pt/C–2 m H_2_SO_4_. Consistent with the OCV trend, the maximum power density was highest for PMo_12_–Pt/C–2 m H_2_SO_4_ at 149.4 mW cm^−^^2^, substantially outperforming PMo_12_–Pt/C (119.6 mW cm^−^^2^), PMo_12_–2 m H_2_SO_4_ (81.8 mW cm^−^^2^), and PMo_12_ (64.6 mW cm^−^^2^). The data shown in Figure 1 clearly demonstrate that incorporating oxidative degradation catalysts into the conventional PMo_12_‐based electrolyte markedly enhances the decomposition of rice straw, accompanied by substantial electron generation. This enhancement is strongly correlated with the facilitated cleavage of diverse C─O and C─C bonds intrinsic to the rice straw matrix. As shown in Figure S5, rice straw comprises a complex matrix of lignin, cellulose, and hemicellulose, in which C─O and C─C bonds are expected to be selectively cleaved depending on the catalyst used during oxidative degradation.

### 2.2 Mechanisms of Bond Cleavage in Rice Straw Components Decomposed by Catalyst

The rice straw used in this study consists of crystalline cellulose at its core, surrounded by hemicellulose and lignin (Figure 2a) [5, 32, 33]. As previously reported, the bulky structure of lignin in unrefined biomass makes the decomposition of rice straw particularly difficult [34, 35, 36]. Lignin contains various types of C─C and C─O linkages, with β–O–4 linkages accounting for approximately 50%, biphenyl for 25%, β‐5 for 10%, and other minor linkages comprising the remaining ∼15% [37, 38]. The bond dissociation energies (BDEs) of these linkages vary depending on the type of bond, and particularly, C─C bonds found in β–1, β–O–4, α–1, biphenyl, and β–5 structures exhibit high BDEs (around 70 to 130 kcal mol^−^^1^), making them more resistant to decomposition [39, 40]. To understand the breakage of these bonds and the formation of the highly reduced PMo_12_ solution, we performed 2D HSQC NMR and ^1^ ^3^C solid‐state NMR analysis on two distinct components: the reduced PMo_12_ solution and biomass decomposition residues that were filtered from the solution. The reduced electrolyte was analyzed using a mixture of D_2_O and H_2_O at 1:9 ratio, while the biomass residue was dissolved in DMSO‐d_6_ with ball‐milling process.

**FIGURE 2:**
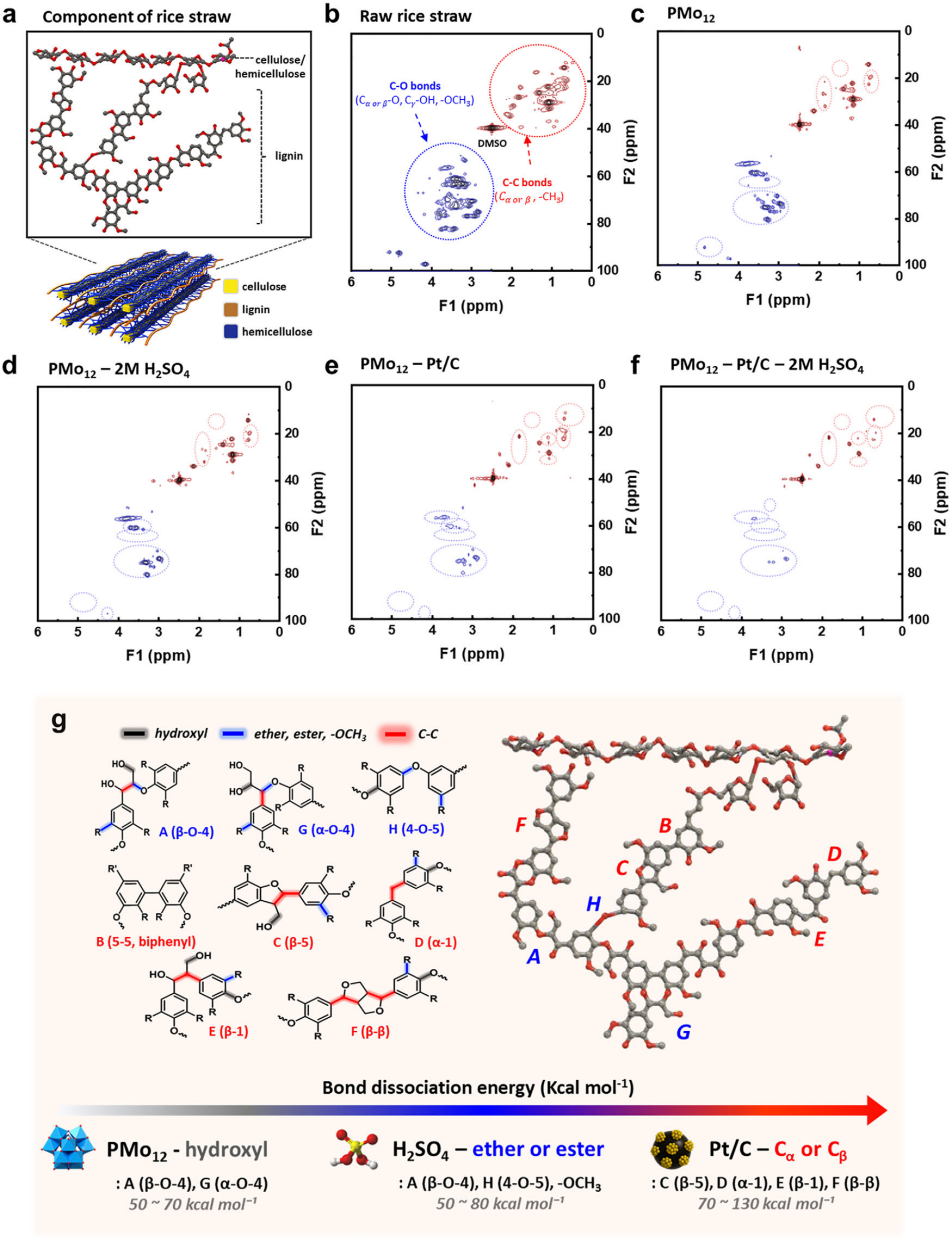
Mechanistic elucidation of selective bond cleavage in rice straw components via catalytic oxidative degradation. (a) Components and specific chemical structure of raw rice straw (lignocellulose). (b) ^1^H–^1^ ^3^C 2D HSQC NMR analysis of raw rice straw. (c‐f) ^1^H–^1^ ^3^C 2D HSQC NMR analysis of degraded residues after treatment with (c) PMo_12_, (d) PMo_12_–2 m H_2_SO_4_, (e) PMo_12_–Pt/C, and (f) PMo_12_–Pt/C–2 m H_2_SO_4_ at 100°C for 12 h. (more detailed bond assignment is denoted at Supporting Figures) (g) Chemical structures of lignin components and proposed role of each catalyst in rice straw degradation by PMo_12_ (gray label: hydroxyl group majorly decomposed by PMo_12_, blue label: ether or ester group decomposed by assist of H_2_SO_4_, red label: carbon‐carbon bonds decomposed by assist of Pt/C). Colors are applied selectively to representative bonds rather than all instances to maintain visual clarity. The corresponding numerical bond dissociation energy (BDE) ranges for each cleavage type are indicated at the bottom.

Based on the 2D NMR data, analysis of the pretreated raw rice straw confirmed the presence of various carbon linkages within the diverse structures of lignocellulose (Figure 2b and Figure S6). The aromatic regions did not show clear signals because raw biomass was used, resulting in low peak intensity in both 2D NMR and ^1^ ^3^C solid‐state NMR. Upon decomposition in the PMo_12_ solution, the breakdown of easily decomposable C─O bonds—such as α–O–4 (50 kcal mol^−^^1^) and β–O–4 (68 kcal mol^−^^1^)—was observed as their disappearance in the NMR spectra compared to the raw rice straw data. Moreover, some C─C bonds—such as alkyl C in β–β (66 kcal mol^−^^1^)—were partially cleaved by PMo_12_ (Figure 2c and Figure S7). When sulfuric acid was applied, additional C─O bond signals disappeared, including methoxy groups (─OCH_3_) and even 4–O–5 (80 kcal mol^−^^1^), while the C─C bond region remained intact (Figure 2d and Figure S8). The more resistant C─C bonds, including β–1 (E, 70 kcal mol^−^^1^), β–O–4 (A, 80 kcal mol^−^^1^), α–1 (D, 90 kcal mol^−^^1^), β–β (F‐1, 66–123 kcal mol^−^^1^), biphenyl (B, 120 kcal mol^−^^1^), and β–5 (C, 130 kcal mol^−^^1^) structures, were cleaved in the presence of Pt/C (Figure 2e and Figure S9). These results clearly corroborate the difference in reduction behavior between sulfuric acid (1‐, 2‐electron reduction) and Pt/C (2‐, 4‐electron reduction) observed in Figure 1, as well as the resulting difference in electrochemical performance. When both sulfuric acid and Pt/C were introduced, most of the linkages were effectively cleaved, with only minor signals corresponding to biphenyl, β–5, and α–1 structures remaining (Figure 2f and Figure S10).

^1^ ^3^C solid‐state NMR further supported these observations, **allowing us to distinguish** the effects of each reaction condition on biomass degradation (Figure S11). In the case of simple PMo_12_ treatment, the breakdown of C_aro_–OH (∼100 ppm) and C_γ_─OH (∼70 ppm), such as those in α–O–4 or β–O–4 structures, was clearly evident. When sulfuric acid was added to the reaction, the NMR data showed that most C─O bonds—including the C_aro_–OCH_3_ methoxy group (50–75 ppm) and the C_α_─O bond (∼80 ppm), even in the 4–O–5 structure—were efficiently decomposed. These results indicate that PMo_12_, assisted by sulfuric acid, is capable of breaking down numerous bonds in lignin structures, except for recalcitrant C─C bonds. Even in the presence of sulfuric acid, certain C─C bonds—particularly those connected to C_α_ or C_β_ in β–1, α–1, biphenyl, and β–5 structures—remained resistant to decomposition. This resistance was overcome when Pt/C was introduced, which significantly enhanced the cleavage of these C─C bonds. This explains why the PMo_12_ anode electrolyte containing Pt/C achieved a **highly reduced state** and exhibited improved performance. Comprehensive analysis using both 2D NMR and ^1^ ^3^C solid‐state NMR enabled us to identify which structural units of rice straw were cleaved and to clarify the distinct role of each catalyst in the degradation process (Figure 2g). The monomeric structures derived from lignocellulose were labeled alphabetically (compound A: β–O–4, B: biphenyl, C: β–5, D: α–1, E: β–1, F–1 and F–2: β–β, G: α–O–4, and H: 4–O–5), and each label was color‐coded to indicate the catalyst responsible for bond cleavage. In summary, PMo_12_ alone was capable of oxidizing or hydrolyzing only limited C─O bonds, primarily hydroxyl‐containing structures (compounds A, G, and F‐2). Sulfuric acid facilitated the hydrolysis of ether and ester linkages, particularly in compounds A and H. Notably, the Pt/C catalyst enabled the efficient cleavage of more recalcitrant C─C bonds, including those in compounds B, C, D, E, and F‐1, resulting in extensive breakdown of the lignocellulosic matrix into smaller molecular fragments.

The 2D NMR analysis of the PMo_12_ anode electrolyte further confirmed these results. In the PMo_12_ solution, the NMR spectra showed that only some of the C─O bonds in the rice straw composition (e.g., cellulose, hemicellulose, and lignin) were cleaved while others remained intact (Figure S12), and this bond cleavage pattern was already confirmed in previous research [34]. When sulfuric acid was applied as the solvent, the spectra in the C─O bond region (F1: 50–100 ppm and F2: 2.5–6.0 ppm) became simplified and new peaks emerged, indicating that the biomass structure had broken down into smaller units (Figure S13). Upon the addition of Pt/C, the decomposition process reached completion, as evidenced by the disappearance of most of the original signals (Figure S14). In the PMo_12_–Pt/C–2 m H_2_SO_4_, the spectra showed only faint signals corresponding to simple monomeric units, confirming that the biomass components were almost completely decomposed, as observed in both the solution and the biomass residue (Figure S15).

### 2.3 Sustainability of the BFFC System

To evaluate the stability of Pt/C during the oxidative degradation of rice straw, repeated degradation reactions were conducted without replacing the Pt/C catalyst, and changes in reduction degree and power density were monitored with respect to the number of cycles. As detailed in the Methods section, to strictly evaluate the stability of the solid catalyst independently, a fresh, newly prepared PMo_12_ solution was used for each cycle. After each reaction, the solid residues containing the Pt/C catalyst were filtered, washed, and added to the new PMo_12_ solution. In Figure 3a,b, the reduction degree of PMo_12_ remained nearly constant regardless of the number of cycles, indicating that the catalytic activity of Pt/C was well maintained throughout repeated use. After each cycle, the average particle size and Pt peak profile of the Pt/C catalyst were analyzed using X‐ray diffraction (Figure S16). No significant change was observed in the Pt particle size—calculated by Scherrer's equation—or in the overall Pt diffraction peaks, confirming the excellent structural stability of Pt/C under the oxidative degradation conditions of rice straw. During the repeated experiments, undecomposed rice straw residues remained associated with the Pt/C catalyst and were co‐filtered along with it (Figure S17). Furthermore, to directly verify the structural integrity of the catalyst at the nanoscale, transmission electron microscopy and EDS mapping were performed on both the fresh and reused Pt/C. As shown in Figures S18 and S19, the TEM images clearly confirm that the ∼2 nm Pt nanoparticles remained uniformly dispersed on the carbon support without any severe aggregation or microscopic biomass fouling, even after repeated exposure to the highly acidic and oxidative reaction matrix. Notably, the EDS mapping of the reused Pt/C detected trace amounts of Mo originating from the PMo_12_ electrolyte. This elemental signature indicates that the PMo_12_ molecules successfully accessed and intimately interacted with the Pt/C active sites during the reaction, effectively facilitating the catalytic electron transfer. This robust morphological stability directly corroborates the sustained structural integrity observed in the XRD patterns (Figure S16) and fully supports the highly stable catalytic reusability.

**FIGURE 3:**
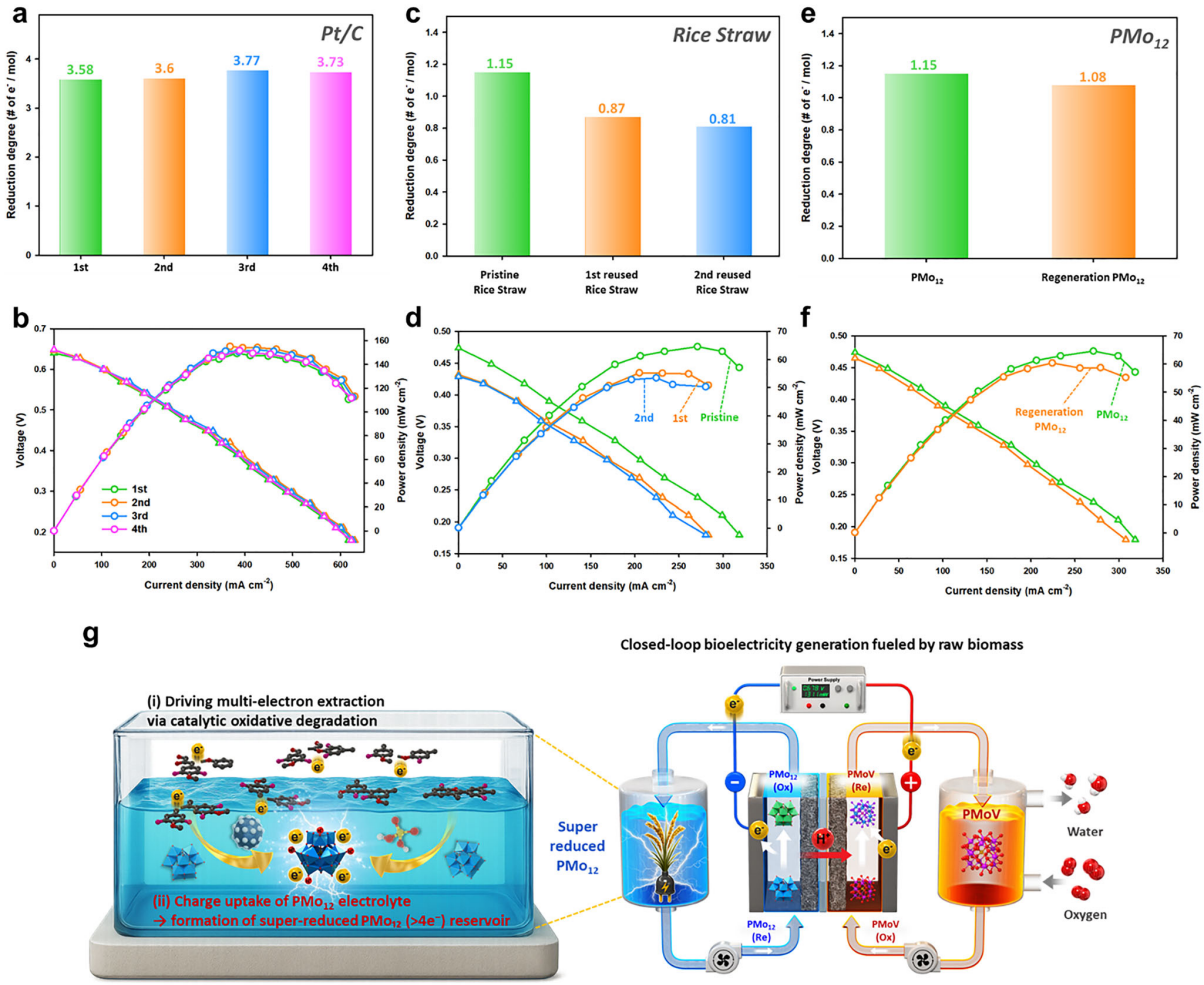
Sustainability assessment via catalyst reusability and closed‐loop electrolyte regeneration. (a) Reduction degree of PMo_12_ over repeated oxidative degradation cycles using a fixed Pt/C catalyst in PMo_12_–Pt/C–2 m H_2_SO_4_. (b) i–V polarization and power density curves of the BFFC during each cycle with the same Pt/C catalyst. (c) Reduction degree of reused rice straw (first and second cycles) in PMo_12_‐control. (d) i–V polarization and power density curves of the BFFC using raw and reused rice straw as fuel. (e) Reduction degree of PMo_12_‐regeneration prepared by reusing discharged PMo_12_ solution. (f) i–V polarization and power density curves of the BFFC using PMo_12_ regeneration. (g) A schematic representation of closed‐loop bioelectricity generation driven by the biomass conversion process in the BFFC system.

To evaluate the fuel efficiency of the residual rice straw, raw rice straw and rice straw subjected to the first and second oxidative degradation cycles were reacted in the fresh PMo_12_‐control solution (without Pt/C), and the reduction degree and power density were measured. These samples were denoted as raw rice straw, first reused rice straw, and second reused rice straw, respectively. The amount of each type of rice straw was fixed at 2 g (Figure 3c,d). The reduction degrees of the reused rice straw were lower than those of the raw rice straw, with 0.87 for the first reused and 0.81 for the second reused samples, compared to 1.15 for the raw rice straw. However, it is noteworthy that the undecomposed bonds in the residual rice straw can undergo further cleavage through repeated oxidative degradation. This supports the results of the Pt reuse experiment (Figure 3a,b), where the slight increase in reduction degree suggests that the residual rice straw did not block the active sites of platinum but instead contributed additionally as fuel.

To further examine the sustainability of the PMo_12_ electrolyte, rice straw was oxidatively degraded in the PMo_12_‐control solution, after which the reduced PMo_12_ was fully discharged (Figure S20). A new electrolyte was then prepared by conducting an additional oxidative degradation of rice straw using the discharged PMo_12_ solution, which was denoted as PMo_12_‐regeneration. As shown in Figure 3e,f, PMo_12_‐regeneration had a reduction degree of 1.08, close to that of the PMo_12_‐control, with a similarly efficient BFFC performance. These results demonstrate that BFFC technology, which directly utilizes raw biowaste as fuel, eliminates the need for costly refining processes and enables continuous operation with reusable feedstocks. Furthermore, this approach provides a simplified and cost‐effective pathway for power generation, underscoring its promise as a sustainable energy solution for the future (Figure 3g).

### 2.4 Scalable Electricity Generation from Raw Rice Straw in the BFFC SYSTEM

To maximize the performance of the BFFC system, the oxidative degradation of rice straw was conducted under varying particle sizes and reaction temperatures. The reaction was carried out in the PMo_12_–Pt/C–2 m H_2_SO_4_ electrolyte by decreasing the rice straw particle size from 250 to 100 µm and increasing the reaction temperature from 100°C to 150°C. While achieving a deep multi‐electron reduction state in polyoxometalates typically relies on applied electrical bias or highly active external reducing agents, our synergistic catalytic system successfully overcame this fundamental thermodynamic barrier relying solely on the chemical energy of spontaneous thermochemical biomass oxidation. Under these conditions, a reduction degree of 4.5 was achieved. This signifies the successful capture of over four electrons per cluster without the application of any external electrical bias, thereby defining the formation of a ‘super‐reduced’ PMo_12_ electrolyte in the context of biomass flow fuel cells (Figure S21). When rice straw was degraded under conditions of 100 µm particle size and 150°C, the resulting PMo_12_ electrolyte enabled the BFFC to achieve a power density of 200.1 mW cm^−^^2^, which represents a 1.34‐fold increase compared to that obtained under the conventional condition of 250 µm and 100°C. In addition to catalyst and fuel optimization, the ion‐exchange membrane also plays a pivotal role in determining BFFC performance. When a thinner Nafion membrane (N211) was employed in place of the conventional N115, a further enhancement in BFFC performance was observed. The maximum power density reached 218.6 mW cm^−^^2^, representing the highest value reported to date among BFFC systems over the past several years (Figure 4a,b and Table S1) [20, 41]. These results are attributed to the enhanced oxidative degradation of rice straw under reduced particle size and elevated temperature, as well as the reduced ohmic and charge transfer resistances achieved by employing the thinner N211 membrane (Figure S22).

**FIGURE 4:**
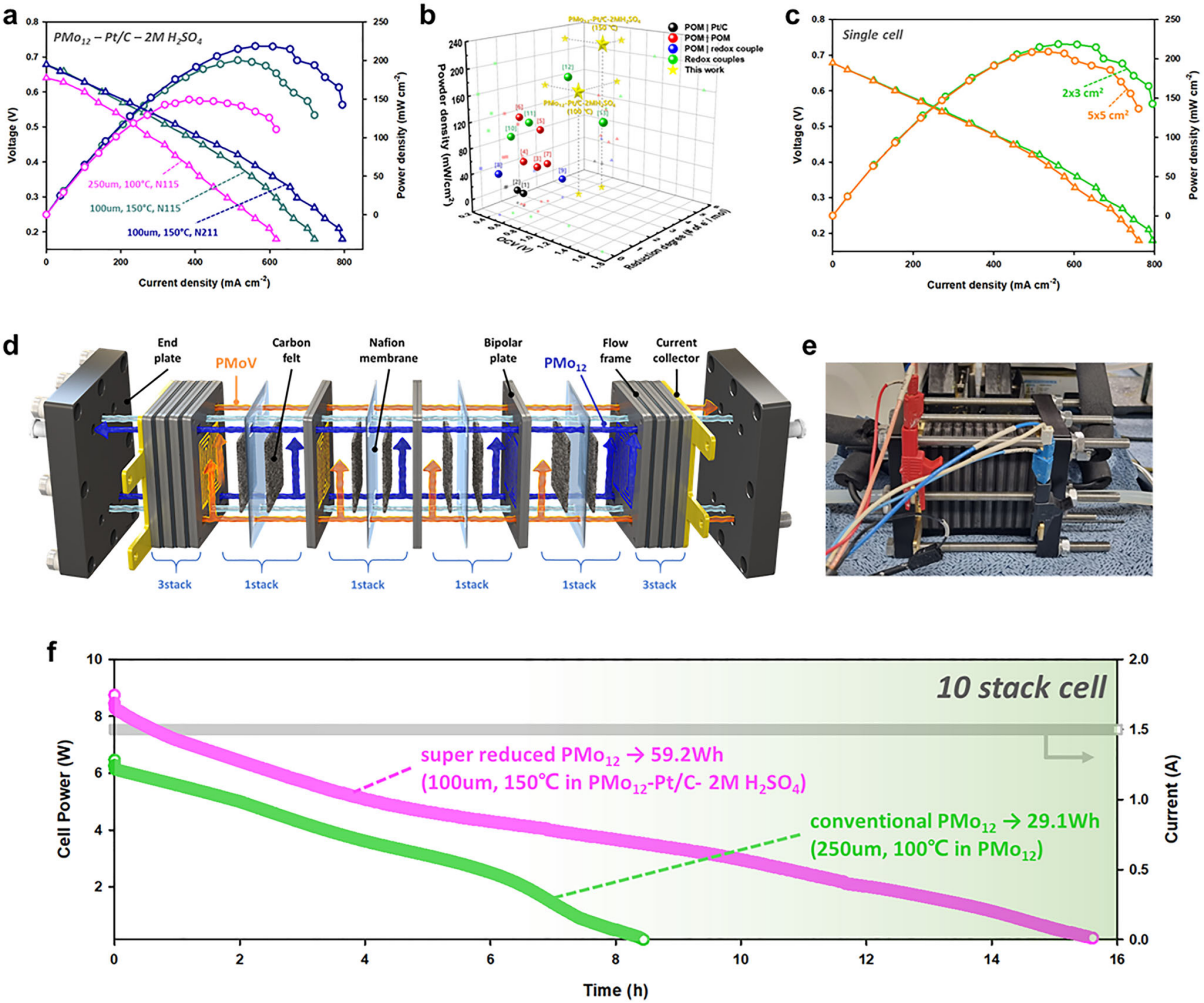
High‐performance and scalable bioelectricity generation in BFFC fueled by raw rice straw. (a) i–V polarization and power density curves of the BFFC in PMo_12_–Pt/C–2 m H_2_SO_4_ under various conditions (particle size, reaction temperature, and membrane type). (b) Comparison of maximum power densities reported in BFFC systems. (c) i–V polarization and power density curves of the 25 cm^2^ single cell. (d) Schematic diagram of the BFFC 10‐stack prototype (active area: 25 cm^2^ × 10 cells, 250 cm^2^). (e) Photograph of the BFFC 10‐stack cell. (f) Discharge profiles of the two electrolytes prepared under conventional and super‐reduced conditions.

To demonstrate the capability of raw rice straw for large‐scale power generation, a 25 cm^2^ single cell was fabricated. Rice straw was decomposed under the maximum power conditions—100 µm particle size and 150°C in PMo_12_–Pt/C–2 m H_2_SO_4_ electrolyte. Under these conditions, the cell achieved a power density of 209.6 mW cm^−^^2^, confirming that comparable performance to the 6 cm^2^ cell can be maintained even at larger scales (Figure 4c). With the exception of adjusting the flow rate from 30 mL min^−^^1^ to 150 mL min^−^^1^ in proportion to the electrode area, all other operating conditions were maintained the same. The scalability of the BFFC system was further examined through the design and fabrication of a prototype reactor consisting of ten stacked 25 cm^2^ cells (Figure 4d,e). The anode electrolytes were prepared by oxidatively degrading rice straw under two different conditions: 250 µm rice straw at 100°C in PMo_12_ (conventional condition) and 100 µm rice straw at 150°C in PMo_12_–Pt/C–2 m H_2_SO_4_ (super‐reduced condition), using 2 L of solution in each case. The cathode electrolyte was consistently maintained as 2 L of PMoV solution. During the continuous stack operation, the PMoV catholyte was oxidatively regenerated by continuously bubbling oxygen gas in the cathode tank. As extensively demonstrated in previous well‐established studies [16], this standard continuous O_2_‐driven regeneration protocol efficiently maintains the PMoV mediator in a highly oxidized state, facilitating stable cathodic reduction and allowing the system to fully leverage the massive electron reservoir generated at the anode. The stack was composed of ten cell assemblies, each consisting of a current collector, bipolar plate, flow frame, air‐treated carbon felt, and an N211 membrane (Figures S23 and S24). The successful operation of the BFFC system is demonstrated in Video S1. Upon discharge, the super‐reduced PMo_12_ electrolyte delivered a total power of 59.2 Wh over 15.6 h—more than twice that of the PMo_12_ electrolyte prepared under conventional conditions, which yielded 29.1 Wh over 8.4 h (Figure 4f). It is noteworthy that the actual charge output of the stack was ∼23.4 Ah. Compared to the massive theoretical stored charge of the super‐reduced anolyte (∼72 Ah, initial reduction degree of 4.5), this corresponds to a Coulombic (electron utilization) efficiency of ∼32.5%, indicating that only about 1.5 electrons per PMo_12_ molecule were utilized after discharge. While this study successfully overcame the biological recalcitrance to unlock a massive electron reservoir at the anode, the full utilization of this capacity in a continuous stack requires equally rapid and matched oxidation/reduction kinetics at the PMoV cathode. Therefore, future engineering focused on enhancing the continuous O_2_‐driven regeneration kinetics of the cathode compartment will be essential to fully unlock this theoretical capacity and further maximize the overall stack utilization efficiency.

This work establishes a practical and efficient approach for direct electricity generation from raw biowaste in the BFFC system, without the need for purification. By optimizing catalytic conditions and reaction parameters, the POM‐based system achieved high electron transfer efficiency and power performance. The successful scale‐up to a multi‐cell prototype further demonstrates the system's potential for future industrial implementation in sustainable biowaste‐to‐energy conversion technologies.

## 3 Conclusion

This study demonstrates a high‐power, scalable BFFC system capable of utilizing raw rice straw waste through the formation of a super‐reduced PMo_12_ electrolyte. Our mechanistic investigation, supported by 2D HSQC and solid‐state NMR analyses, confirmed that the synergistic catalytic system effectively cleaves recalcitrant C─C linkages. By dynamically scavenging electrons from the Pt/C surface to prevent catalyst deactivation, this system unlocks a multi‐electron storage capacity (>4e^−^) previously inaccessible in biomass electrolytes. Crucially, we translated this fundamental chemical insight into a practical engineering breakthrough. The successful operation of the 10‐cell stack prototype, delivering 59.2 Wh, represents a significant leap from laboratory‐scale experiments to realistic energy applications. Furthermore, the demonstrated reusability of the catalyst and the regeneration of the electrolyte underscore the potential for a sustainable, closed‐loop material cycle. This work suggests that by coupling catalytic oxidative degradation with scalable flow cell engineering, agricultural waste can be transformed from an environmental burden into a reliable source of carbon‐neutral electricity. We anticipate that this platform will pave the way for distributed bio‐energy systems that are both high‐performing and practically deployable.

## Author Contributions

**H. Lee**: Conceptualization, Data curation, Methodology, Formal analysis, Writing – original draft. H.‐J. Lee: Data curation, Methodology, Formal analysis, Writing – original draft. J. Lee, S.‐S. Han, S. Park: Data curation, **Resources. E. S. Cho**: Writing – review & editing. **H.‐T. Kim**: Conceptualization, Supervision, Funding acquisition, Project administration, Writing – review & editing.

## Conflicts of Interest

The authors declare no conflicts of interest.

## Supporting information




**Supporting File 1**: advs78361‐sup‐0001‐SuppMat.docx.

**Supporting File 2**: advs78361‐sup‐0002‐VideoS1.mp4.

## Data Availability

The data that support the findings of this study are available from the corresponding author upon reasonable request.
