# Supplementary material for: Synergistic Charge Scavenging in Pt/C‐Polyoxometalate Systems for Scalable Biomass Power Generation via Super‐Reduced Electrolytes
Source: Adv Sci (Weinh). 2026 Oct 25:e78361. Online ahead of print. doi: 10.1002/advs.78361 (PMC13696770; doi:10.1002/advs.78361)
Supplement: Supplementary file 1 [file ADVS-9999-e78361-s002.docx]

**Supplemental Information**

Synergistic Charge Scavenging in Pt/C-Polyoxometalate Systems for Scalable Biomass Power Generation via Super-Reduced Electrolytes

Hojin Lee^1,2^, Hyeong-Jin Lee^1^, Jongseo Lee^3^, Sang-Soo Han^3^, Sangjin Park^3^, Eun Seon Cho^1, *^ and Hee-Tak Kim^1, *^

^1^Department of Chemical and Biomolecular Engineering, Korea Advanced Institute of Science and Technology (KAIST), Daejeon 34141, Republic of Korea

^2^Clean Energy Research Center, Korea Institute of Science and Technology (KIST), Hwarang-road 14-gil 5, Seongbuk-gu, Seoul 02792, Republic of Korea

^3^3rd R&D Institute, Agency for Defense Development, Daejeon 34186, South Korea

H. Lee and H.-J. Lee contributed equally to this work.

**Correspondence to:** Hee-Tak Kim (heetak.kim@kaist.ac.kr)

**Table of Contents**

Methods (page 3-7)

Supplementary Figures S1-28 (page 8-36)

Supplementary Table S1 (page 37)

Supplementary References (page 38-40)

**Methods**

**Preparation of reduced PMo₁₂**

As biomass feedstocks, two types of fuel were employed: rice straw as an unrefined biomass fuel and lignin as a refined fuel. In particular, rice straw required additional pretreatment. It was freeze-dried for 24 h to obtain an accurate biomass mass and to facilitate its subsequent crushing. The dried rice straw was mechanically ground using a blender (Philips 5000-series HR3575/70) operating at 22,000 rpm, and then sieved using fine mesh sieves (100 μm / 250 μm). For the general preparation of the anode electrolyte, phosphomolybdic acid hydrate (54.758 g, 0.03 mol) was added to 100 mL of deionized (DI) water to form a 0.3 mol·L⁻¹ POM solution. Biomass (2 g) was then added to the POM solution. This mixture was heated at the desired temperature for 12 h using a heating mantle to obtain the reduced anode electrolyte. The resulting slurry was then subjected to vacuum filtration. (**Figure S25**). During this process, POMs accept electrons and are reduced, with the number of electrons acquired increasing according to the extent of oxidative degradation (**Figure S26**). To prepare the super-reduced solution, 100 mL of 2 M H₂SO₄ was used as the solvent instead of DI water, and Pt/C (1 g) was added to the POM solution. To evaluate the reusability of Pt/C, the reacted solution was filtered, and the residues were washed thoroughly with DI water and dried in a vacuum oven for 24 h. These dry residues, which contain Pt/C powder, were mixed with biomass (2 g) in a 0.3 mol·L⁻¹ PMo₁₂ solution, and this mixture was subjected to the same treatment to prepare the reduced PMo₁₂ solution. To investigate the recyclability of the rice straw residues, the above biomass degradation–filtration–washing–drying process was repeated multiple times, and these biomass residues (2 g) were reused for the preparation of the reduced PMo₁₂ solution. For regeneration of the anode electrolyte, the reduced electrolyte was fully discharged in the anode of the BFFC. Subsequently, biomass (2 g) was added to the discharged solution, which was then reheated under the same conditions to regenerate the reduced anolyte.

**Preparation of PMoV**

The cathode electrolyte was synthesized by the same method from Odyakov’s work [14, 15]. A typical synthesis process includes three stages: (1) preparation of PV_14_ solution (solution A), (2) preparation of PMo solution (solution B), and (3) mixture of PMoV solution (cathode POM). Preparation of solution A: V₂O₅ (25.48 g) was added in 1500 mL of DI water and stirred in an ice bath (~4 °C) for 30 minutes. 30wt% H₂O₂ (200 mL) was poured into the mixture and stirred for additional 30 minutes. 85wt% H₃PO₄ (2.66 mL) was added to decompose any extra H₂O₂ under stirring at room temperature for 4 h. Preparation of solution B: MoO₃ (103.68 g) was added into DI water (1000 mL) and stirred for 1 h. 85wt% H₃PO₄ (5.66 mL) was poured into the solution and then heated to boiling until a yellow solution formed. Synthesis of cathode POM solution: 200 mL of solution A was gradually poured into solution B, and the mixture was kept boiling until a homogeneous solution had formed. Water was evaporated to the desired volume, and the concentration was adjusted to 0.3 mol·L⁻¹.

**Characterization**

The changes in **chemical bonds within the biomass** before and after the reaction were analyzed by **2D ¹H–¹³C** heteronuclear single quantum coherence **(HSQC) nuclear magnetic resonance (NMR)** spectroscopy using a 700 MHz spectrometer. The degraded samples were separated into two types: **(i) the filtrate**, which contained both fractionated biomass and POMs in an aqueous phase (Type A), and **(ii) the retained residues**, which were washed several times with deionized water (Type B). For NMR analysis, the filtrate (Type A) was mixed with H₂O and D₂O in a 1:1 ratio. **Meanwhile**, pristine biomass and the **retained** residues (Type B) were dissolved by ball-milling them in DMSO-d₆ with 8 wt% LiCl.
Cross-polarization magic angle spinning ¹³C solid-state NMR (CP-MAS ¹³C SSNMR) was used to investigate changes in carbon-based bonds using the following parameters: a rate of 25 kHz, cross polarization time (1.1 ms), d1 (5 sec), number of signals (1 k), time-domain (8 k), Fourier transform (8 k), line broadening (50 Hz). The average reduction degree was first estimated via **KMnO₄ redox titration**, followed by UV–Vis spectroscopy to obtain a linear correlation between reduction degree and UV–Vis absorbance. UV–Vis measurements were performed using a Thermo Genesys 10S UV–Vis spectrophotometer with a standard quartz cuvette with a 10 mm path length. To ensure exact quantitative reproducibility and to avoid detector saturation by the highly reduced and deeply colored 0.3 M PMo_12_, all post-reaction samples were filtered through a 0.2 µm nylon filter and subsequently diluted according to a rigorous dual-wavelength strategy. Initially, the samples were heavily diluted (e.g., by a factor of 1/30,000 to reach ~10 µM) and measured at 208 nm (corresponding to the O → Mo charge transfer band) to accurately determine the exact total POM concentration in the solution, accounting for any potential volume changes during the reaction (**Figure S27**). Based on this calibrated concentration, the samples were precisely diluted to a specific level (e.g., by a factor of 1/3,000 to reach ~100 µM) and measured at 700 nm (the intervalence charge transfer band between reduced Mo species) to calculate average reduction degree.

To precisely evaluate the donor-acceptor charge transfer gaps, the obtained UV–Vis absorption spectra were converted into Tauc plots. The absorption coefficient (α) was calculated from the absorbance data, and the optical bandgap energy (E_g_) was determined using the Tauc relation for direct allowed transitions: (αhν)² = B (hν – E_g_), where hν is the incident photon energy and B is an energy-independent constant. The E_g_ values for each sample were extracted by linearly extrapolating the steepest portion of the (αhν)² versus hν curves to the energy axis (where α = 0). The number of reduced electrons was also determined using **³¹P NMR with a 500 MHz spectrometer**. X-ray diffraction (PXRD) was carried out using a Rigaku SmartLab High-Resolution Powder X-ray Diffractometer to identify the crystalline structure and particle size of the catalysts.

**Single-cell performance tests and electrochemical analysis**

The BFFC cell was assembled using a Nafion membrane, carbon felts, flow frames, bipolar plates, Au-coated copper current collectors, inlet-outlet plates, and end plates. The electrode consisted of 4-mm-thick carbon felt (SGL Carbon, Germany) that had been heat-treated at 500 °C in air for 9 hours, with an active area of 6 cm² for both the positive and negative electrodes. The electrodes were compressed by 20% during cell assembly. A Nafion membrane (NRE 115, 211) was placed between the positive and negative electrodes to serve as a separator (**Figure. S28).** Cell tests were performed with 50 mL of prepared electrolyte supplied to both the positive and negative compartments. The PMo₁₂-based anode electrolyte and the PMoV cathode electrolyte were pumped through the BFFC at a flow rate of 30 mL min⁻¹. The PMoV cathode electrolyte was regenerated by continuously bubbling oxygen gas in the cathode tank. The entire BFFC system was operated at 80 °C. The i–V polarization curves were measured in voltage step mode, varying the cell voltage in 0.03 V increments from the open-circuit voltage (OCV) down to 0.18 V. Electrochemical impedance spectroscopy (EIS) measurements were conducted at OCV over a frequency range from 0.1 Hz to 100 kHz to evaluate ohmic and charge transfer resistance.

**Fabrication of the BFFC 10-stack prototype**

The custom-designed 250 cm² BFFC stack consisted of air heat-treated carbon felts (5 × 5 cm²), Nafion 211 membranes, flow frames, bipolar plates, current collectors, and end plates. These components were assembled into ten stacked modules to construct the 250 cm² BFFC stack system. For high-power output, 2 L of super-reduced PMo₁₂ anode electrolyte (prepared by oxidative degradation of 100 μm rice straw at 150 °C in PMo₁₂–Pt/C–2 M H₂SO₄) and 2 L of PMoV cathode electrolyte were pumped through the BFFC stack cell at a flow rate of 150 mL min⁻¹. The PMoV cathode electrolyte was regenerated by bubbling oxygen gas in the cathode tank. The BFFC stack system was operated at 80 °C. To maintain the system temperature, a circulator was used to continuously flow 80 °C water through both the water jacket and the stack cell. To preserve electrolyte concentration, another circulator was used to circulate 2 °C ethanol for reflux. Discharge testing was performed at a constant current of 1.5 A using a VSP potentiostat (BioLogic).

**Supplemental Results**

**Figure S1.** **(a)** UV–Vis spectra of PMo₁₂ after 12 h degradation using commercial lignin and raw rice straw as fuels. **(b)** Reduction degree of PMo₁₂ after 12 h degradation using commercial lignin and raw rice straw as fuels. **(c)** i–V polarization (left y-axis, triangles) and power density (right y-axis, circles) curves of the BFFC fueled by commercial lignin and raw rice straw. **(d)** Open-circuit voltage (OCV) and maximum power density of the MEAs.

**Figure S2.** Full-range UV–Vis absorption spectra of the reduced PMo₁₂ electrolytes. The spectra were obtained after the catalytic oxidative degradation of raw rice straw in PMo₁₂, PMo₁₂-2 M H₂SO₄, PMo₁₂-Pt/C, and PMo₁₂-Pt/C-2 M H₂SO₄.

**Figure S3.** The solubility change of PMo₁₂ in different H₂SO₄ concentrations. PMo₁₂ dissolves in ~2.0 M of H₂SO₄, while it precipitates in 2.5 M.

**Figure S4.** Evaluation of the intrinsic reduction degrees of individual biomass components and model monomers. (a) Bar chart comparing the average reduction degree achieved by reacting the baseline 0.3 M PMo₁₂ solution with individual biopolymers (cellulose, hemicellulose, lignin) and their corresponding monomers (glucose, xylose, coniferyl alcohol). (b) ³¹P NMR spectra of the PMo₁₂ solutions after reacting with hemicellulose, xylose, and coniferyl alcohol, confirming the generation of 4-electron reduced PMo₁₂ species.

To elucidate the specific origins of the deep multi-electron reduction and evaluate the electron-transfer selectivity of different biomass constituents, control experiments were conducted using purified biopolymers and representative model monomers. To purely assess their intrinsic thermodynamic capacity to donate electrons to the POM, the reactions were performed under the baseline PMo₁₂ condition (100 °C, without Pt/C and H₂SO₄), thereby excluding kinetic artifacts or aggressive parasitic side reactions (e.g., hyper-volatilization or repolymerization) that occur under extreme catalytic environments.

As shown in Figure S4a, the highly crystalline cellulose and heavily cross-linked kraft lignin exhibited low reduction degrees of 0.5 and 0.4, respectively, confirming their recalcitrance against unassisted PMo₁₂ oxidation. While monomeric sugars such as glucose (1.4 e^-^) and xylose (2.5 e^-^) displayed moderate electron-donating capacities, coniferyl alcohol (a representative lignin-derived phenolic monomer) reached 4.2 e^-^. To spectroscopically validate these macroscopic titration results, ³¹P NMR analysis was conducted on the reacted solutions (Figure S4b). Crucially, the spectra of the PMo₁₂ solutions reacted with hemicellulose, xylose, and coniferyl alcohol all explicitly exhibited the characteristic peak corresponding to the 4-electron reduced PMo₁₂ species. This direct spectroscopic evidence aligns with the reduction degree values and unequivocally proves that these specific components—especially, the xylan/xylose moieties from hemicellulose and the p-π conjugated phenolic moieties from lignin—act as the primary high-capacity electron donors. Furthermore, the stark contrast between polymeric lignin (0.4 e^-^) and its monomeric counterpart (4.2 e^-^) highlights the strict necessity of the Pt/C catalyst to cleave robust C–C bonds and liberate these high-potential donors from the raw biomass matrix.

**Figure S5.** The structural illustration of lignocellulose (cellulose, hemicellulose, lignin) which represents rice straw structure, and summarized redox process of PMo₁₂-Pt/C-2M H₂SO₄ during biomass depolymerization reaction.

**Figure S6.** ¹H–¹³C -HSQC 2D NMR spectra of raw rice straw. Generally, the blue letters indicate C-O bonds and the red letters indicate C–C bonds.

**Figure S7.** ¹H–¹³C -HSQC 2D NMR spectra of degraded rice straw by PMo₁₂. After the degradation, the signals of the blue letter region disappear, which are generally hydroxyl groups present in compound **A**, **F-2**, **G** structures.

**Figure S8.** ¹H–¹³C -HSQC 2D NMR spectra of degraded rice straw by PMo₁₂-2M H₂SO₄. After the degradation, the signal of blue letter region disappears further, which also includes ether and ester groups present in compound **A**, **H**, and **-OCH_3_**.

**Figure S9.** ¹H–¹³C -HSQC 2D NMR spectra of degraded rice straw by PMo₁₂-Pt/C. After the degradation, the signals of both blue and red letter regions disappear, which include C–C bonds in compound **C**, **D**, **E**, and **F**.

**Figure S10.** ¹H–¹³C -HSQC 2D NMR spectra of degraded rice straw by PMo₁₂-Pt/C-2M H₂SO₄. After the degradation, the signals of blue letters are almost absent and the signal of red letter region disappears more intensely, indicating that almost all C–O and C–C bonds in lignin were decomposed.

**Figure S11.** ¹³C solid-state NMR of raw rice straw and degraded residues after biomass oxidation. The green letter indicates aromatic C bonds, the blue letter indicates C-O bonds, and the red letter indicates C–C bonds in lignin structure.

**Figure S12.** ¹H–¹³C -HSQC 2D NMR spectra of anode electrolyte PMo₁₂ after biomass degradation.

**Figure S13.** ¹H–¹³C -HSQC 2D NMR spectra of anode electrolyte PMo₁₂-2M H₂SO₄ after biomass degradation.

**Figure S14.** ¹H–¹³C -HSQC 2D NMR spectra of anode electrolyte PMo₁₂-Pt/C after biomass degradation.

**Figure S15.** ¹H–¹³C -HSQC 2D NMR spectra of anode electrolyte PMo₁₂-Pt/C-2M H₂SO₄ after biomass degradation.

**Figure S16.** Intact PXRD crystallinity and the average particle size of Pt/C after each biomass degradation by PMo₁₂-Pt/C-2M H₂SO₄.

**Figure S17**. Photograph of degraded rice straw and remaining Pt/C residues after repeated catalytic oxidative degradation experiments.

**Figure S18**. High-resolution transmission electron microscopy (HR-TEM) image (left) and corresponding energy-dispersive X-ray spectroscopy (EDS) elemental mapping (right) of the fresh Pt/C catalyst.

**Figure S19**. High-resolution transmission electron microscopy (HR-TEM) image (left) and corresponding energy-dispersive X-ray spectroscopy (EDS) elemental mappings (middle and right) of the reused Pt/C catalyst recovered after repeated oxidative degradation cycles.

**Figure S20.** UV–Vis spectra of pristine and fully discharged PMo₁₂ at BFFC. The nearly identical spectral profiles indicate complete consumption of the reduced electrons.

**Figure S21.** UV–vis spectra and the average reduction degree of anode electrolyte PMo₁₂-Pt/C-2M H₂SO₄.

**Figure S22.** The impedance analysis changing rice straw substrates (e.g. 100 μm and 250 μm), reaction temperature (e.g. 100 °C and 150 °C), and Nafion membranes (e.g. N115 and N211).

**Figure S23.** Photograph of 10-stack cell components for long-term BFFC test.

**Figure S24.** Photograph of overall BFFC system with the continuous circulation of anode and cathode electrolytes (e.g. PMo₁₂ and PMoV).

**Figure S25.** Pre-treatment of raw biomass (e.g. rice straw) before depolymerization and thermal-reduction process of PMo₁₂ electrolyte.

**Figure S26.** Linear relationship between peak intensity at 700 nm and the average reduction degree of reduced PMo₁₂ electrolyte. The reduction degree was varied by the change of reaction temperature and time. The corresponding linear regression equation is as follows: Reduction degree = 3.8232 * peak intensity at 700nm + 0.1241 (R² = 0.9888).

**Figure 27**. Calibration curve for determining the precise concentration of PMo12 using UV-Vis spectroscopy. The plot exhibits a highly linear correlation (R2 = 0.9825) between the PMo12 concentration and the peak intensity at 208 nm (O → Mo charge transfer band). The accompanying table summarizes the exact concentration values and their corresponding measured peak intensities used to derive the linear regression equation.

**Figure S28.** Photograph of individual single cell components for BFFC test.

**Table S1**. Comparison table of the average reduction degree, open circuit voltage, and power density among reported biomass flow fuel cell systems.^1-13^

**References**

1. Liu, W., Mu, W., Liu, M., Zhang, X., Cai, H., and Deng, Y. Solar-induced direct biomass-to-electricity hybrid fuel cell using polyoxometalates as photocatalyst and charge carrier. Nat Commun *5*, 3208, doi:10.1038/ncomms4208 (2014).

2. Zhao, X., and Zhu, J.Y. Efficient Conversion of Lignin to Electricity Using a Novel Direct Biomass Fuel Cell Mediated by Polyoxometalates at Low Temperatures. ChemSusChem *9*, 197-207, doi:10.1002/cssc.201501446 (2016).

3. Liu, W., Mu, W., Deng, Y. High-Performance Liquid-Catalyst Fuel Cell for Direct Biomass-into-Electricity Conversion. Angew. Chem. Int. Ed. 53, 49, 13558-13562, doi:10.1002/anie.201408226 (2014).

4. Zhang, Z., Liu, C., Liu, W., Du, X., Cui, Y., Gong, J., Guo, H., and Deng, Y. Direct conversion of sewage sludge to electricity using polyoxomatelate catalyzed flow fuel cell. Energy *141*, 1019-1026, doi:10.1016/j.energy.2017.09.143 (2017).

5. Liu, C.M., Zhang, Z., Liu, W., Xu, D., Guo, H., He, G.L., Li, X.M., and Deng, Y.L. Flow fuel cell powered by combustible agricultural waste. Clean Energy *2*, 20-28, doi:10.1093/ce/zky001 (2018).

6. Zhang, Z., Liu, C.M., Liu, W., Cui, Y., Du, X., Xu, D., Guo, H., and Deng, Y.L. Innovative design of coal utilization - A green pathway for direct conversion of coal to electricity through flow fuel cell technology. Applied Energy *200*, 226-236, doi:10.1016/j.apenergy.2017.05.073 (2017).

7. Liu, W., Gong, Y.T., Tricker, A., Wu, G.X., Liu, C.M., Chao, Z.S., and Deng, Y.L. Fundamental Study toward Improving the Performance of a High-Moisture Biomass-Fueled Redox Flow Fuel Cell. Industrial & Engineering Chemistry Research *59*, 4817-4828, doi:10.1021/acs.iecr.9b06982 (2020).

8. Ding, Y., Du, B., Zhao, X., Zhu, J. Y., Liu, D. Phosphomolybdic acid and ferric iron as efficient electron mediators for coupling biomass treatment to produce bioethanol and electricity generation from wheat straw. Bioresource Technology 228, 279-289, doi:10.1016/j.biortech.2016.12.109 (2017).

9. Yang, H.S., Bai, Y.C., Ouyang, D.H., Wang, F.Q., Liu, D.H., and Zhao, X.B. Coupling biomass pretreatment for enzymatic hydrolysis and direct biomass-to-electricity conversion with molybdovanadophosphoric heteropolyacids as anode electron transfer carriers. Journal of Energy Chemistry *58*, 133-146, doi:10.1016/j.jechem.2020.09.009 (2021).

10. Gong, J., Liu, W., Du, X., Liu, C., Zhang, Z., Sun, F., Yang, L., Xu, D., Guo, H., and Deng, Y. Direct Conversion of Wheat Straw into Electricity with a Biomass Flow Fuel Cell Mediated by Two Redox Ion Pairs. ChemSusChem *10*, 506-513, doi:10.1002/cssc.201601441 (2017).

11. Zu, X., Sun, L., Gong, J., Liu, X., Liu, Y. Ferric ion pair mediated biomass redox flow fuel cell and related chemical reaction kinetics study. Chem. Eng. J. 348, 476-484, doi:10.1016/j.cej.2018.03.190 (2018).

12. Ouyang, D., Wang, F., Hong, J., Gao, D., Zhao, X. Ferricyanide and vanadyl (V) mediated electron transfer for converting lignin to electricity by liquid flow fuel cell with power density reaching 200 mW/cm^2^. Appl. Energy 304, 117927, doi:10.1016/j.apenergy.2021.117927 (2021).

13. Xie, Z., Zu, X., Lin, J., Qiu, X., Liang, T., Chen, L. A high-performance lignin flow fuel cell based on self-generating electricity of lignin at low temperature via a privileged structure and redox chemistry. Green Chem. 26, 2021-2030, doi:10.1039/D3GC04428A (2024).

14. Odyakov, V. F., Zhizhina, E. G., and Maksimovskaya, R. I. Synthesis of molybdovanadophosphoric heteropoly acid solutions having modified composition. Appl. Catal. A-Gen. 342, 126-130, doi: 10.1016/j.apcata.2008.03.008 (2008).

15.Gunn, N. L. O., Ward, D. B., Menelaou, C., Herbert, M. A., and Davies, T. J. Investigation of a chemically regenerative redox cathode polymer electrolyte fuel cell using a phosphomolybdovanadate polyoxoanion catholyte. Journal of Power Sources 348, 107-117, doi: 10.1016/j.jpowsour.2017.02.048 (2017).
